# Tangshen Formula Alleviates Hepatic Steatosis by Inducing Autophagy Through the AMPK/SIRT1 Pathway

**DOI:** 10.3389/fphys.2019.00494

**Published:** 2019-04-26

**Authors:** Yan Wang, Hailing Zhao, Xin Li, Nan Li, Qian Wang, Yanzhen Liu, Qionglin Liang, Zixing Shao, Nannan Zhang, Tingting Zhao, Liang Peng, Ping Li

**Affiliations:** ^1^Beijing Key Laboratory for Immune-Mediated Inflammatory Diseases, Institute of Clinical Medical Sciences, China-Japan Friendship Hospital, Beijing, China; ^2^Graduate School of Peking Union Medical College, Chinese Academy of Medical Science & Peking Union Medical College, Beijing, China; ^3^Beijing University of Chinese Medicine, Beijing, China; ^4^Key Laboratory of Bioorganic Phosphorus Chemistry and Chemical Biology (Ministry of Education), Department of Chemistry, Tsinghua University, Beijing, China

**Keywords:** Tangshen formula, fatty liver, AMPK, SIRT1, autophagy

## Abstract

Tangshen formula (TSF), a formula of Chinese herbal medicine, improves lipid metabolism in humans and animals with diabetic kidney disease. However, the effect and mechanism of TSF on nonalcoholic fatty liver disease (NAFLD) remain unclear. The activation of autophagy appears to be a potential mechanism for improving NAFLD. In the present study, we examined the therapeutic effect of TSF on hepatic steatosis and sought to explore whether its effect is related to activating autophagy. Here, we showed that TSF treatment significantly attenuated hepatic steatosis in both high-fat diet (HFD) and methionine choline-deficient diet (MCDD)-fed mice. Meanwhile, TSF reduced lipid accumulation in palmitate (PA)-stimulated HepG2 cells and primary mouse hepatocytes. Furthermore, TSF increased Sirtuin 1 (SIRT1) expression and promoted autophagy activation *in vivo*. TSF also improved PA-induced suppression of both SIRT1 expression and SIRT1-dependent autophagy, thereby alleviating intracellular lipid accumulation *in vitro*. In addition, TSF increased SIRT1 expression and induced autophagy in an adenosine monophosphate-activated protein kinase (AMPK)-dependent manner. Moreover, SIRT1 knockdown abolished the autophagy-inducing and lipid-lowering effects of TSF. In conclusion, TSF improved lipid accumulation and hepatic steatosis by inducing the AMPK/SIRT1 pathway-mediated autophagy.

## Introduction

Nonalcoholic fatty liver disease (NAFLD) has become a worldwide health concern due to the increased incidence of obesity and diabetes. In addition, NAFLD is closely associated with the risk factors of coronary heart disease, such as metabolic syndrome, diabetes mellitus, and dyslipidemia, which are considered to be the leading causes of death (Wiest et al., 2017). Although our understanding of the pathogenesis of NAFLD has significantly improved, there is still no effective medication for this disease that is approved by FDA (Neuschwander-Tetri et al., 2015). Thus, development of new therapeutic strategies capable of alleviating NALFD is urgently needed.

Lipid homeostasis is maintained by the balance between lipid synthesis and lipid metabolism, and the latter is critical for the progression of hepatic steatosis (Zhang et al., 2015). Autophagy is a genetically programmed, evolutionarily conserved catabolic process that promotes cell survival and modulates intracellular homeostasis (Piekarski et al., 2018). Autophagy has many other pathophysiological functions in lipid metabolism aside from removing intracellular pathogens and damaged cell organelles, such as preventing hepatic steatosis by facilitating the hydrolysis of intracellular lipids, as found in recent studies (Liu et al., 2015; Huang et al., 2017). After being activated by starvation or rapamycin, autophagy breaks more stored lipids and expedites subsequent free fatty acid β-oxidation in the mitochondria (Chen et al., 2016; He et al., 2016). In contrast, when autophagy is abrogated by the knockout of autophagy-related genes (ATGs), lipids accumulate in the hepatocytes, which leads to the development of hepatic steatosis (Weidberg et al., 2009; Madrigal-Matute and Cuervo, 2016). As impaired hepatic autophagy aggravates the development of hepatic steatosis, the induction or restoration of hepatic autophagy might facilitate the clearance of intracellular lipids and prevent NAFLD development.

Sirtuin 1 (SIRT1), an important member of the Sir2 family, plays a pivotal role in preventing hepatic metabolic damage by modulating autophagy (Song et al., 2015). The activation of SIRT1-protein induction-mediated autophagy is an important protective mechanism against hepatic steatosis in caloric restriction, which is the most effective intervention for NAFLD progression to date (Kim et al., 2016). In addition, pharmacological upregulation of SIRTI expression promotes lipid droplet hydrolysis in autolysosomes and suppresses hepatic lipid accumulation in ob/ob mice (Hong et al., 2018). SIRT1 is regulated by highly mixed interactions with multiple unrelated targets in which adenosine monophosphate-activated protein kinase (AMPK) plays a vital role. In response to energy depletion or stress conditions, phosphorylated AMPK (p-AMPK) increases SIRT1 expression, thus triggering autophagy to regulate energy homeostasis and metabolic stress (Sun et al., 2018).

The Tangshen formula (TSF) is based on empirical evidence and is mainly used for the clinical treatment of diabetic kidney diseases (DKD). In our preliminary multicenter, randomized, double-blind, and placebo-controlled trial, TSF significantly reduced proteinuria, improved the estimated glomerular filtration rate (eGFR) among patients with DKD, and exhibited a beneficial effect in improving plasma lipid metabolism (Yu et al., 2011; Li et al., 2015). Additional studies from our group also found that TSF not only exerted a renal protective effect in decreasing urinary albumin excretion rate and glomerulosclerosis but also improved lipid metabolism in spontaneous DKD rodents, including Otsuka Long-Evans Tokushima Fatty rats and db/db mice (Zhang et al., 2011; Kong et al., 2016; Liu et al., 2018). However, little is known about the effect and potential mechanism of TSF on hepatic steatosis. Considering our previous finding that TSF induces renal autophagy and the critical role of autophagy in hepatic steatosis progression (Zhao et al., 2017), we aimed to explore the effect of TSF on hepatic steatosis and investigate the underlying mechanism by assessing the potential roles of autophagy and the AMPK/SIRT1 pathway, in particular.

## Materials and Methods

### Herbal Formulation and Components

TSF was extracted from the following seven natural herbs: astragalus root [*Astragalus membranaceus* (Fisch.) Bge.], burning bush twig [*Euonymus alatus* (Thunb.) Sieb.], rehmannia root (*Rehmannia glutinosa* Libosch.), bitter orange (*Citrus aurantium* L.), cornus fruit (*Cornus officinalis* Sieb. et Zuce), rhubarb root and rhizome (*Rheum palmatum* L.), and notoginseng root [*Panax notoginseng* (Burk.) F. H. Chen] in the ratio of 10:5:4:3.4:3:2:1 (W/W), respectively, based on the dry weight of the product. TSF was prepared and standardized by Jiangyin Tianjiang Pharmaceutical Co. according to the established guidelines in the Pharmacopeia of the People’s Republic of China 2010. The chemical composition of TSF was verified as described previously (Zhao et al., 2017), and the six most common compounds in TSF (loganin, calycosin-7-O-β-D-glucoside, naringenine-7-rhamnosidoglucoside, neohesperidin, naringenin, and aloe-emodin) were identified.

### Animals and Experimental Design

**FIGURE 1:**
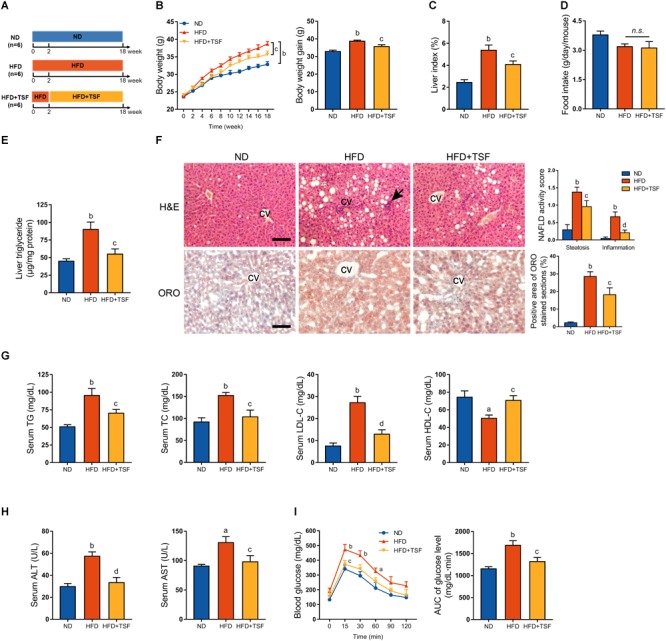
TSF alleviated hepatic steatosis in mice fed a HFD. **(A)** Male mice fed a HFD for 18 weeks and administered TSF (2.4 g/kg/day) by gavage for 16 weeks. **(B,C)** Body weight was recorded every week, and body weight gain and food intake were measured at the 18th week. **(D)** Liver index was calculated as the ratio of liver weight to body weight (%). **(E)** Liver triglycerides were measured. **(F)** Hepatic steatosis and inflammatory cells infiltration (black arrow) in H&E- and ORO-stained sections of mice fed a HFD treated with or without TSF, bar = 50 μm; CV represents central vein; NAFLD activity score (hepatic steatosis and lobular inflammation) and positive Oil Red O staining area. **(G)** Serum TG, TC, LDL-C, HDL-C, ALT, and AST of mice fed a HFD treated with or without TSF. **(H)** After overnight fasting, IPGTT was measured at 0, 15, 30, 60, 90, and 120 min; AUC was subsequently calculated. **(I)** Serum ALT and AST of mice fed a HFD treated with or without TSF. Data from each group are expressed as the mean ± SEM (*n* = 6).^a^*P* < 0.05, ^b^*P* < 0.01 vs. ND group; ^c^*P* < 0.05, ^d^*P* < 0.01 vs. HFD group.

Eight weeks old C57BL/6J mice weighing 23–25 g were obtained from Beijing HFK Bioscience (China). The mice subjected to high-fat diet (HFD) were randomly divided into two groups (*n* = 6 per group) after 1 week of acclimation: HFD group and HFD with TSF (HFD + TSF) group; a third normal diet (ND) group was included as control. The ND and HFD groups were fed with chow diet or HFD (containing 60% kcal from fat) for 18 continuous weeks. The mice in the HFD + TSF group were fed with a HFD for 2 weeks, after which they were gavaged with TSF (2.4 g/kg/day) for 16 weeks (Figure 1A). The mice in the ND and HFD groups were gavaged with the same volume of distilled water.

**FIGURE 2:**
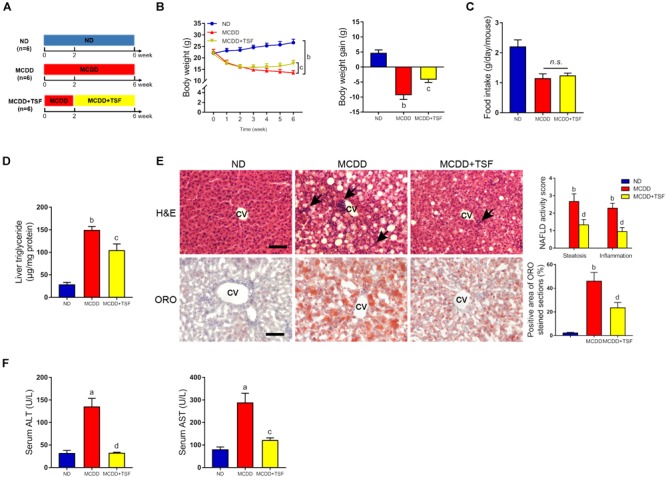
TSF alleviated hepatic steatosis in mice fed a MCDD. **(A)** Male mice fed a MCDD for 6 weeks and administered TSF (2.4 g/kg/day) by gavage for 4 weeks. **(B,C)** Body weight was recorded every week, and body weight gain and food intake were measured at the 6th week. **(D)** Liver triglycerides were measured. **(E)** Hepatic steatosis and inflammatory cells infiltration (black arrow) in H&E- and Oil Red O-stained sections of mice fed a MCDD treated with or without TSF, bar = 50 μm; CV represents central vein; NAFLD activity score (hepatic steatosis and lobular inflammation) and positive Oil Red O staining area. **(F)** Serum ALT and AST of mice fed a MCDD treated with or without TSF. Data from each group are expressed as the mean ± SEM (*n* = 6).^a^*P* < 0.05, ^b^*P* < 0.01 vs. ND group; ^c^*P* < 0.05, ^d^*P* < 0.01 vs. MCDD group.

Similarly, the mice subjected to methionine choline-deficient diet (MCDD) were randomly divided into two groups after 1 week of acclimation (*n* = 6 per group), MCDD and MCDD with TSF (MCDD + TSF) groups; a third ND group was included as control. Mice in the ND and MCDD groups were fed with normal chow diet or MCDD (containing 21% kcal from fat without L-methionine or choline bitartrate) for 6 continuous weeks, whereas those in the MCDD + TSF group were fed with MCDD for 2 weeks, after which they were gavaged with TSF (2.4 g/kg/day) for an additional 4 weeks (Figure 2A). The mice belonging to the ND and MCDD groups were gavaged with the same volume of distilled water. All mice were housed at 20–25°C and 65–75% humidity using a 12 h light/dark cycle and received chow and water *ad libitum*. The animals were weighed once a week, and food intake was measured at the 18th or 6th week. Subsequently, all mice were sacrificed after overnight fasting by anesthesia using pentobarbital sodium. Serum and liver samples were collected rapidly for follow-up measurements. This study was carried out in accordance with the recommendations in the Guide for the Care and Use of Laboratory Animals of the National Institutes of Health. The protocol was approved by the Ethics Committee of the China–Japan Friendship Hospital.

### Cell Culture and Treatment

Human hepatoma HepG2 cells were purchased from the American Type Culture Collection (Manassas, VA, United States). Primary mouse hepatocytes were isolated from mice via collagenase IV perfusion through the inferior vena cava, as described previously (Tomita et al., 2017), and were placed on petri dishes coated with 0.1% collagen I. The cells were cultured in Dulbecco’s Modified Eagle’s Medium supplemented with 10% fetal bovine serum at 37°C in a humidified atmosphere containing 5% CO_2_. Cellular steatosis was induced with 0.3 mM palmitate (PA; P9767, Sigma-Aldrich, St. Louis, MO, United States) for 24 h, and equal amounts of fatty acid-free bovine serum albumin (BSA) were added to the control cells. After successfully producing the steatosis model, the cells were incubated in a series of concentrations (25, 50, 100 μg/mL) of TSF for an additional 24 h with PA (0.3 mM). In addition, the cells were treated with PA (0.3 mM) for 24 h to induce steatosis, followed by incubation with TSF (100 μg/mL) and chloroquine (CQ; 20 μM; C6628, Sigma-Aldrich, St. Louis, MO, United States) or bafilomycin A1 (BafA1; 10 nM; C6628, Sigma-Aldrich, St. Louis, MO, United States). Steatotic HepG2 cells were exposed to compound C (CC; 10 μM; S730, Selleck Chemicals, Houston, TX, United States) with TSF (100 μg/mL) for 24 h. Complete culture medium was used in all experiments to avoid starvation-induced autophagy. PA was dissolved in fatty acid-free BSA as described previously (Hur et al., 2015).

### Cell Viability Detection

Cells were plated into 96-well plates and incubated for 24 h to allow cell adherence. Then, the culture medium was replaced with DMEM supplemented with PA (0.3 mM) and TSF (0, 25, 50, 100, 200, and 400 μg/mL). After 24 h of incubation, cell viability was determined by colorimetry using the 3-(4,5-dimethylthiazol-2-yl)-2,5-diphenyltetrazolium bromide (MTT). Insoluble formazan crystals were dissolved in dimethyl sulfoxide (DMSO) and measured at 490 nm with a Thermo microplate reader (Thermo Fisher Scientific, Waltham, MA, United States).

### Cell Transfection of siRNA

HepG2 cells were transfected with nontargeting small interfering RNA (siRNA) and siRNA targeting SIRT1 (purchased from GenePharma, Shanghai, China) using Lipofectamine 3000 (Invitrogen, Carlsbad, CA, United States) according to the manufacturer’s protocol. The siRNA targeting human SIRT1 sequences were used: sense, 5′-CCCUGUAAAGCUUUCAGAAdtdt-3′, and antisense, 5′-UUCUGAAAGCUUUACAGGGdtdt-3′.

### Intracellular Lipid Droplets

Accumulation of intracellular lipid droplets was detected by BODIPY 493/503 staining. Cells (2 × 10^3^ cells/well) were seeded into 96-well culture plates and incubated for 24 h to allow for cell adherence. After induction of the steatotic cells with PA (0.3 mM) and treatment with or without TSF (100 μg/mL) and CQ (20 μM), the cells were fixed with 4% buffered paraformaldehyde and stained with BODIPY 493/503 working solution (1 μg/mL) for 15 min at 37°C. Images and integrated fluorescence intensity were quantified using ImageXpress Micro XLS Widefield High Content Screening System.

### Autophagy Flux Assay Using Fluorescent-Tagged Light Chain Microtubule-Associated Protein 3 (LC3)

HepG2 cells were infected with red fluorescent protein (RFP)-green fluorescent protein (GFP)-LC3 adenovirus followed by incubation with or without PA (0.3 mM) and TSF (100 μg/mL). After the fusion of the autophagosomes and lysosomes, an acid-sensitive GFP signal is quenched in the lysosome, whereas the RFP signal is relatively stable. The overlay of RFP-coupled LC3 (RFP-LC3) and GFP-coupled LC3 (GFP-LC3) creates yellow puncta and aid in the identification of the autophagosomes relative to the autolysosomes (remaining red puncta). Images of cells were captured by confocal laser scanning microscopy. The excitation wavelengths of RFP-LC3 and GFP-LC3 were 550–590 nm and 395–495 nm, respectively.

### Cell Electron Microscopy

HepG2 cells were fixed with 2% glutaraldehyde in 0.1 M phosphate buffer (pH 7.4) followed by 1% OsO_4_. After dehydration, thin sections were stained with uranyl acetate and lead citrate for observation, and images were obtained via transmission electron microscopy (JEOL-100CXII, JEOL, Japan). The number of autophagic vacuoles and lipid droplets was counted from HepG2 cells, which were randomly selected from each group.

### Liver Tissue Pathology

A portion of liver tissue fixed with buffered formalin (10%) was sectioned at 5 μm in paraffin sections. Hematoxylin and eosin (H&E) staining was performed as described previously (Li et al., 2015). The NAFLD activity score of H&E staining, which is based on hepatic steatosis and lobular inflammation, was evaluated blindly by two pathologists under 200× power. Hepatic steatosis grade was scored based on steatosis area: 0 (<5%), 1 (5–33%), 2 (33–66%), and 3 (>66%). Lobular inflammation grade was scored focusing on clusters of inflammatory cells in the hepatic lobule: 0 (absent), 1 (<2 foci), 2 (2–4 foci), and 3 (>4 foci) (Liu et al., 2014). A portion of the liver tissue fixed with paraformaldehyde (4%) was sectioned at 8 μm in a cryostat. Cryostat sections were stained with Oil Red O (ORO) dissolved in 70% isopropyl alcohol, and then images were captured under a microscope (Olympus, Tokyo, Japan). The percentage of the positive area for ORO staining was calculated using Image-Pro Plus software (Media Cybernetics, Warrendale, PA, United States).

### Biochemistry Measurements

Serum TG, total cholesterol (TC), low density lipoprotein-cholesterol (LDL-C), high-density lipoprotein-cholesterol (HDL-C), alanine aminotransferase (ALT), and aspartic acid transaminase (AST) levels were determined using an automatic analyzer (Abbott Diagnostics, Abbott Park, IL, United States). The mice were made to fast overnight prior to intraperitoneal injection of glucose (IPGTT) at the 18th week, as described previously (Hong et al., 2018). Tail vein blood was collected, and fasting blood glucose was measured using Accu-Chek (Roche, Mannheim, Germany).

### Western Blot Analysis

Protein lysates (20–50 μg) of liver tissue and cells were separated in 12% sodium dodecyl sulfate gels and then were transferred onto polyvinylidene fluoride membranes. After blocking in 5% BSA in Tris-buffered saline and Tween-20, membranes were incubated with specific primary antibodies, including rabbit p-AMPK (1:1000; 2535S, Cell Signaling Technology, Danvers, MA, United States), rabbit AMPK (1:1000; 2532S, Cell Signaling Technology, Danvers, MA, United States), rabbit SIRT1 (1:1000; 2028S, CST), rabbit LC3B antibody (1:2000; L7543, Sigma-Aldrich, St. Louis, MO, United States), rabbit p62 (1:1000; PM045, MBL International, Des Plaines, IL, United States) and mouse β-actin antibody (1:3000; sc-376421, Santa Cruz Biotechnology, Santa Cruz, CA, United States) overnight at 4°C. After washing with TBST, the membranes were incubated with horseradish peroxidase-conjugated IgG. Blots were developed using ECL Detection Kit (Amersham Pharmacia Biotech, Buckinghamshire, United Kingdom) and then detected using a ChemiDoc XRS system (Bio-Rad, Hercules, CA, United States). Protein bands were quantified using densitometry with Image J (NIH, Bethesda, MD, United States).

### Statistical Analyses

Quantitative data were presented as the mean ± standard error. Comparisons among groups were performed using one-way analysis of variance, and GraphPad Prism software version 6.0 (GraphPad Prism, San Diego, CA, United States) was used for the analysis. *P* < 0.05 and <0.01 were considered statistically significant.

## Results

### TSF Alleviated Hepatic Steatosis in Mice Fed With HFD or MCDD

HFD-stimulated western diet is widely utilized to induce obesity-associated hepatic steatosis and NAFLD in rodents (Mridha et al., 2017). TSF alleviated aberrant body weight gain and reduced the ratio of liver weight to body weight in HFD-fed mice without any impact on food intake, which represented a benefit for TSF in treating NAFLD (Figure 1B–D). The HFD-fed mice exhibited an obvious aberrant increase in liver TG when compared with the ND-fed mice, which was markedly attenuated by TSF gavage (Figure 1E). Consistent with these changes, TSF administration apparently reduced the area of hepatic steatosis; the number of ballooning hepatocytes and infiltrating inflammatory cell clusters was also reduced, thereby partially reversing the hepatic steatotic morphological changes in HFD-fed mice (Figure 1F). With regard to improvements in lipid metabolism, the abnormally increased levels of serum TG, TC, and LDL-C and decreased levels of serum HDL-C were recovered by TSF in mice fed with a HFD (Figure 1G). Additionally, the increase in serum ALT and AST levels caused by impaired hepatocytes and necrosis was attenuated by TSF (Figure 1H).

Given that insulin resistance is a well-known cause of fatty liver disease that results from lipid overconsumption, we adopted IPGTT to evaluate the effect of TSF on glucose metabolism. Area under the curve results demonstrated that TSF significantly ameliorated glucose tolerance in HFD-fed mice (Figure 1I). Taken together, these results indicated that TSF could effectively improve hepatic steatosis and NAFLD-associated symptoms in mice with long-term lipid overconsumption.

To further ensure the therapeutic effects of TSF on NAFLD, we adopted a MCDD-induced nonalcoholic steatohepatitis (NASH) model that rapidly evolves into severe hepatic steatosis and inflammation. MCDD-fed mice treated with TSF showed obvious improvements in typical symptoms of NAFLD, which were consistent with the HFD-fed mice (Figure 2B–D and Supplementary Figures S1A,B). Interestingly, TSF significantly attenuated advanced lobular inflammation and inhibited the rapid increase in serum ALT and AST levels (Figure 2E,F).

### TSF Enhanced Autophagy and Increased AMPK Activation and SIRT1 Expression in the HFD and MCDD Mice Models

**FIGURE 3:**
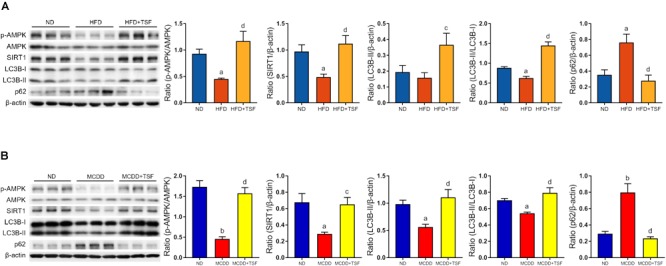
TSF enhanced autophagy and increased AMPK activation and SIRT1 expression in mice fed a HFD or MCDD. **(A)** Western blot assay and semi-quantitative analysis of p-AMPK, AMPK, SIRT1, LC3B-II, p62 expression and the ratio of LC3B-II to LC3B-I in mice fed a HFD treated with or without TSF; data from each group are expressed as the mean ± SEM (*n* = 6) from three repeated western blot experiments of each mouse. **(B)** Western blot assay and semi-quantitative analysis of p-AMPK, AMPK, SIRT1, LC3B-II, and p62 expression in mice fed a MCDD treated with or without TSF; data from each group are expressed as the mean ± SEM (*n* = 6) from three repeated western blot experiments of each mouse. ^a^*P* < 0.05, ^b^*P* < 0.01 vs. ND group; ^c^*P* < 0.05, ^d^*P* < 0.01 vs. MCDD group.

It is generally estimated that long-term continuous lipid challenge suppresses the positive regulatory effects of AMPK and SIRT1 on autophagy in hepatic cells, resulting in low-level autophagy activity and intracellular lipid accumulation (Yao et al., 2018). Detection of autophagy and the expression of autophagy-related regulators in the liver were assayed by western blot (Figure 3A,B). The results of the optical density analysis showed that the ratio of p-AMPK to AMPK and expression of SIRT1, the downstream target of p-AMPK, were decreased in the HFD-fed and MCDD-fed mice when compared with the ND-fed mice, suggesting that the regulatory mechanisms of autophagy were obviously inhibited by lipid accumulation in the liver. Consistently, LC3B-II levels and the LC3B-II/LC3B-I ratio were decreased, whereas p62 levels were significantly increased in the HFD-fed and MCDD-fed mice compared with the ND-fed mice. After TSF treatment, p-AMPK/AMPK ratio and SIRT1 expression were restored in NAFLD mice. Furthermore, TSF administration apparently upregulated LC3B-II levels, the LC3B-II/LC3B-I ratio and reduced p62 levels in mice fed with HFD or MCDD. The results indicated that long-term TSF administration might promote hepatic autophagy through the AMPK/SIRT1 pathway.

### TSF Alleviated Steatosis in PA-Stimulated HepG2 Cells and Hepatocytes

**FIGURE 4:**
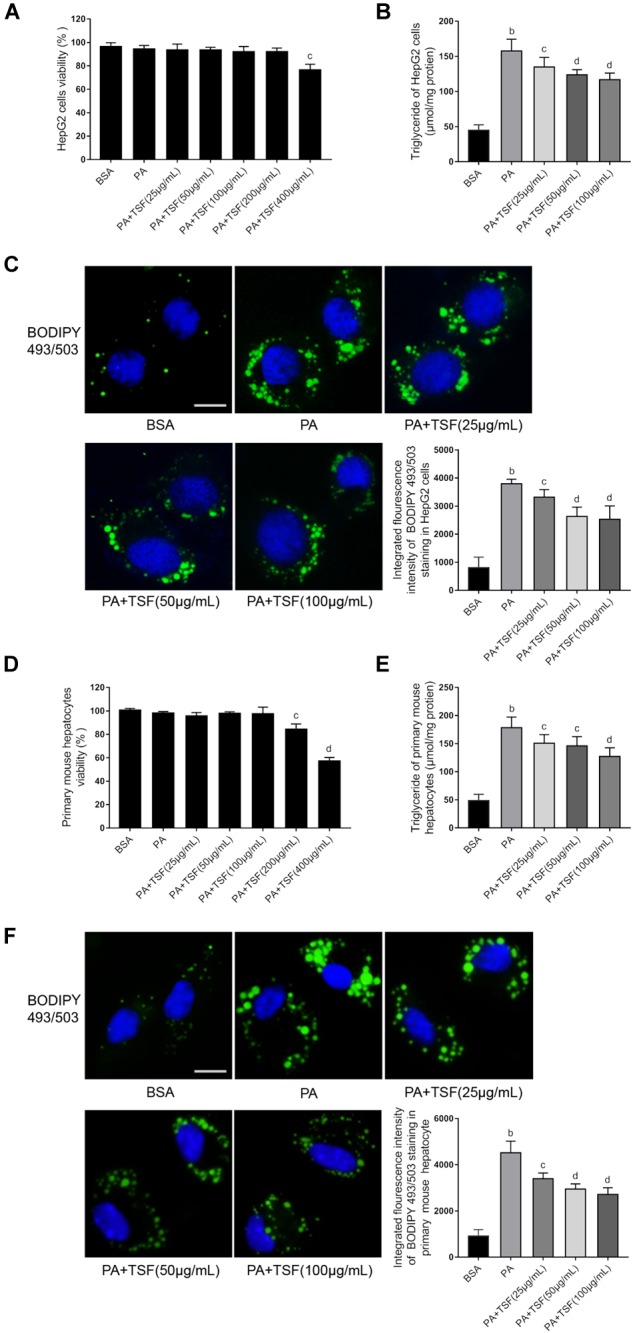
TSF alleviated steatosis in PA-stimulated hepatic cells. **(A,D)** The viability of HepG2 cells and primary mouse hepatocytes was determined using MTT assay; data are expressed as the mean ± SEM of three independent experiments performed in triplicate. **(B,E)** The dose dependence of TSF-reduced induction of intracellular triglyceride in PA-stimulated HepG2 cells and primary mouse hepatocytes; data are expressed as the mean ± SEM of three independent experiments performed in triplicate. **(C,F)** Intracellular lipid droplets of HepG2 cells and primary mouse hepatocytes were stained with BODIPY 493/503; nuclei were stained with DAPI; fluorescent images and intensity data were collected and assessed using a high-content screening system, bar = 5 μm; data are expressed as the mean ± SEM of three independent experiments performed in triplicate. ^b^*P* < 0.01 vs. BSA group; ^c^*P* < 0.05, ^d^*P* < 0.01 vs. PA group.

When incubated with PA, the viability of HepG2 cells and primary mouse hepatocytes was not impacted by 100 μg/mL TSF (Figure 4A,D). After 24 h pretreatment with 0.3 mM PA, the level of intracellular TG and fluorescence intensity of BODIPY 493/503-stained lipid droplets in HepG2 cells and primary mouse hepatocytes were obviously increased, indicating that steatosis was successfully induced (Figure 4B,E). The addition of various concentrations (25, 50, 100 μg/mL) of TSF to the steatotic HepG2 cells and primary mouse hepatocytes reduced the TG content and intensity of BODIPY 493/503 in a dose-dependent manner (Figure 4C,F).

### TSF Alleviated Steatosis by Reinforcing Autophagy in PA-Stimulated HepG2 Cells

**FIGURE 5:**
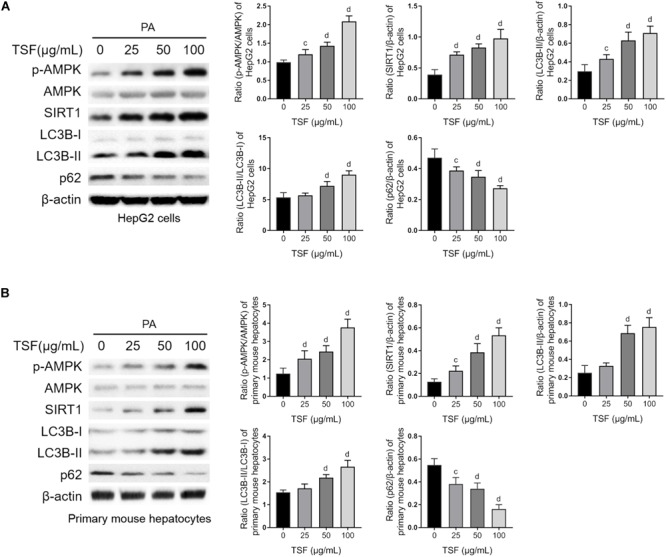
TSF reinforced autophagy dose dependently in PA-stimulated hepatic cells. **(A,B)** The dose dependence of TSF-induced upregulation of the p-AMPK/AMPK ratio, SIRT1 expression, LC3B-II expression and the ratio of LC3B-II to LC3B-I as well as downregulation of p62 in PA-stimulated HepG2 cells and primary mouse hepatocytes determined by western blot assay and semi-quantitative analysis; data are expressed as the mean ± SEM of three independent experiments performed in triplicate.^c^*P* < 0.05, ^d^*P* < 0.01 vs. PA group.

TSF significantly increased SIRT1 expression and the ratio of p-AMPK to AMPK in a dose-dependent manner in steatotic HepG2 cells and primary mouse hepatocytes (Figure 5A,B). Consistent with the above results, TSF increased LC3B-II expression, LC3B-II/LC3B-I ratio, and p62 degradation in a dose-dependent manner, strongly indicating an enhancement in autophagy (Figure 5A,B).

**FIGURE 6:**
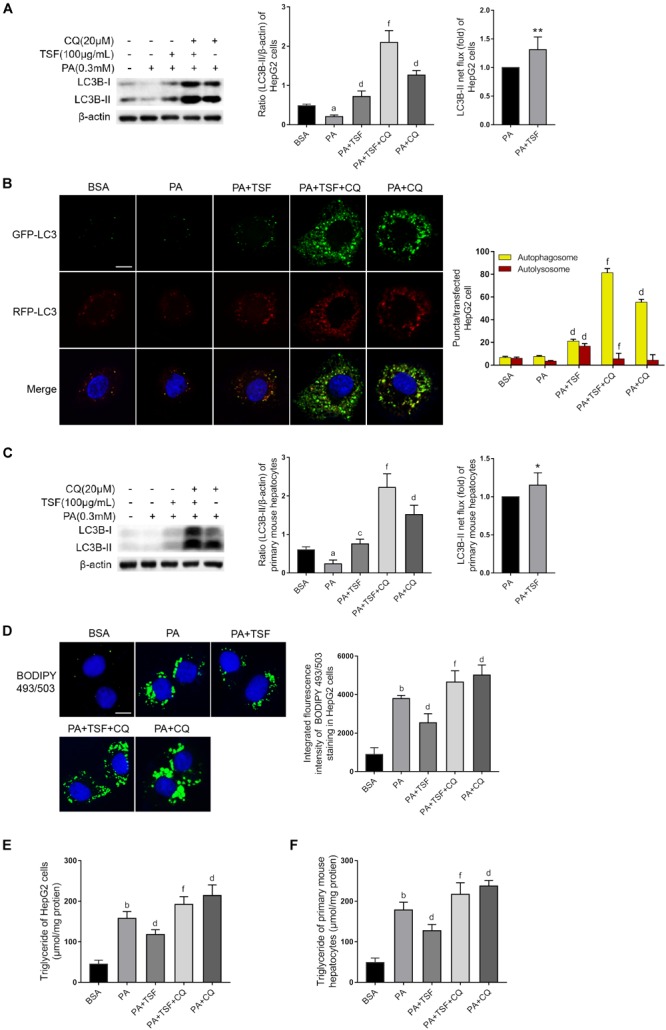
TSF alleviated steatosis through reinforcing autophagy in PA-stimulated hepatic cells. **(A,C)** Western blot assay and semi-quantitative analysis of LC3B-II of PA-stimulated HepG2 cells and primary mouse hepatocytes in response to TSF and CQ treatment for 24 h; LC3B-II net flux was assessed by subtracting the amount of LC3B-II in the absence of CQ from the amount of LC3B-II in the presence of CQ for each of the conditions; each LC3-II expression level was normalized by its β-actin expression level and the LC3B-II net flux of PA-stimulated cells was normalized to 1; data are expressed as the mean ± SEM of three independent experiments performed in triplicate. **(B)** Autophagosomes (yellow puncta) and autolysosomes (red puncta) of PA-stimulated HepG2 cells in response to TSF and CQ treatment for 24 h; nuclei were stained with DAPI; bar = 2.5 μm; the number of autophagosomes and autolysosomes per HepG2 cell (*n* = 30) was counted. **(D)** BODIPY 493/503-stained intracellular lipid droplets of PA-stimulated HepG2 cells in response to TSF and CQ treatment for 24 h; nuclei were stained with DAPI; fluorescent images and intensity data were collected and assessed using a high-content screening system, bar = 5 μm, data are expressed as the mean ± SEM of three independent experiments performed in triplicate. **(E,F)** Intracellular triglycerides in PA-stimulated HepG2 cells and primary mouse hepatocytes in response to TSF and CQ treatment for 24 h, data are expressed as the mean ± SEM of three independent experiments performed in triplicate.^a^*P* < 0.05, ^b^*P* < 0.01 vs. BSA group; ^c^*P* < 0.05, ^d^*P* < 0.01 vs. PA group; ^f^*P* < 0.01 vs. PA+TSF group; ^∗^*P* < 0.05, ^∗∗^*P* < 0.01 vs. PA+CQ group.

CQ is a lysosomotropic base and raises lysosomal pH directly. BafA1 is a lysosomal proton pump inhibitor and interrupts lysosomal acidification. Both CQ and BafA1 are widely used to block the autophagy flux by elevating lysosomal pH, thereby inhibiting the activity of resident lipases and proteases during autophagosome–lysosome fusion. Consequently, cells treated with CQ are unable to hydrolyze lipids and degrade intra-autophagosomal surface, and exhibit lipid accumulation and LC3-II accumulation in the cytoplasm, consistent with a blocked autophagy. In this study, we first used CQ (20 μM) to assess the effect of TSF on autophagy using LC3B-II net flux and transfected HepG2 cells with RFP-GFP-LC3 construct (Kim et al., 2017). CQ or BafA1 further stimulated LC3B-II accumulation and aggravated LC3B-II net flux in PA-stimulated HepG2 cells after treatment with TSF for 24 h when compared with the PA-stimulated HepG2 cells treated with CQ or BafA1 alone (Figure 6A and Supplementary Figure S2). CQ challenge also led to the accumulation of autophagosomes in PA-stimulated HepG2 cells after incubation with TSF (100 μg/mL) for 24 h when compared with the cells treated with PA and TSF only (Figure 6B). Of note, autophagy is a dynamic process, and newly synthesized double-membrane autophagosomes containing lipids, long-lived cellular proteins, or other cytoplasmic contents need to fuse with lysosomes to form single-membrane autolysosome and complete cytoplasmic content degradation by lipases and proteases within autolysosome (Tanaka et al., 2016). In the HepG2 cells, analysis of the autophagy flux of RFP-GFP-LC3 construct revealed that TSF increased both autophagosome (yellow puncta) and autolysosome (red puncta) formation, thus indicating the facilitating roles of TSF in autophagy initiation and flux (Figure 6B). Similar accumulative changes in LC3B-II levels and LC3B-II turnover under CQ challenge were noted in primary mouse hepatocytes (Figure 6C). These results demonstrated that TSF not only strengthened the initiation of autophagy but also promoted autophagy flux in the hepatic cells.

Secondly, to determine whether TSF-induced autophagy activation was involved in the inhibition of steatosis, autophagy flux was pharmacologically blocked with CQ. As expected, CQ interdicted the hydrolysis of lipid droplets in autolysosomes and exacerbated the accumulation of TG and lipid droplets in PA-stimulated HepG2 cells and primary mouse hepatocytes (Figure 6D–F). CQ also counteracted the intracellular lipid-lowering effect induced by TSF, given that no differences in intracellular TG content and lipid droplets were noted between steatotic cells treated with or without TSF under the CQ challenge (Figure 6D–F). These results demonstrated that TSF improved hepatic steatosis by reinforcing autophagy potentially through the AMPK/SIRT1 pathway.

### TSF-Induced Autophagy Was AMPK/SIRT1-Dependent in PA-Stimulated HepG2 Cells

**FIGURE 7:**
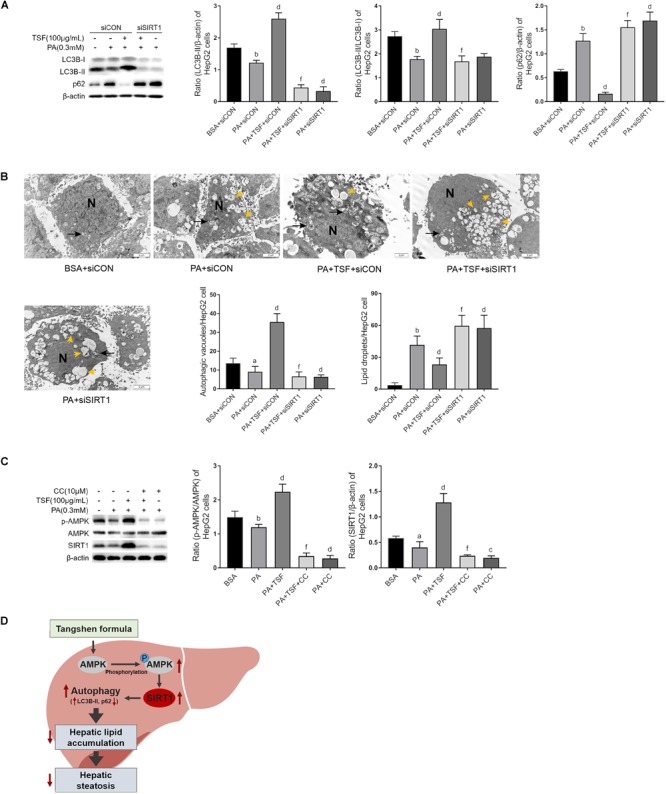
TSF-induced autophagy is AMPK/SIRT1 dependent in PA-stimulated HepG2 cells. **(A)** Knockdown SIRT1 by siRNA abolished TSF-induced upregulation of LC3B-II, the ratio of LC3B-II to LC3B-I and downregulation of p62 in PA-stimulated HepG2 cells, as determined by western blot assay and semi-quantitative analysis, data are expressed as the mean ± SEM of three independent experiments performed in duplicate. **(B)** Knockdown SIRT1 by siRNA abolished TSF-induced upregulation of autophagic vacuoles with double-membrane structure (black arrows) and reduction in lipid droplets (yellow arrows) in PA-stimulated HepG2 cells, as determined by transmission electron microscopy; N represents the nucleus; bar = 2 μm; the number of autophagic vacuoles and lipid droplets per cell (*n* = 30) was counted. **(C)** Inhibiting AMPK phosphorylation via compound C abolished TSF-induced upregulation of SIRT1, as determined by western blot assay and semi-quantitative analysis, data are expressed as the mean ± SEM of three independent experiments performed in duplicate. **(D)** Model of the newly proposed mechanism in whereby TSF signaling alleviates hepatic steatosis by inducing AMPK/SIRT1 pathway mediated autophagy.^a^*P* < 0.05, ^b^*P* < 0.01 vs. BSA group; ^d^*P* < 0.01 vs. PA group; ^f^*P* < 0.01 vs. PA+TSF group.

To determine whether TSF-induced autophagy was associated with SIRT1 induction and AMPK activation, we used specific SIRT1 siRNA to knock down SIRT1 expression and an AMPK inhibitor CC to block AMPK phosphorylation in HepG2 cells. As shown in Figure 7A,B, the upregulation of LC3B-II levels, LC3B-II/LC3B-I ratio, and autophagic vacuoles and the downregulation of intracellular lipid droplets by TSF were notably attenuated following siRNA-mediated SIRT1 knockdown. These findings indicate the involvement of SIRT1 in the autophagy-inducing and lipid-lowering effects of TSF in steatotic HepG2 cells. After the phosphorylation of AMPK was blocked by CC, TSF-induced upregulation of SIRT1 was attenuated (Figure 7C). Thus, we concluded that TSF-induced autophagy and attenuated lipid accumulation by activating the AMPK/SIRT1 pathway.

## Discussion

NAFLD is one of the major causes of chronic liver disease, leading to serious liver-related complications and an increase in the overall mortality rate (Zheng et al., 2018). Effective therapeutic strategies for individuals with NALFD are urgently needed. We have previously demonstrated that TSF improves lipid metabolism in humans and animals with diabetic kidney disease (Yang et al., 2016; Liu et al., 2018). In the present study, we examined the effect of TSF on hepatic steatosis in NAFLD models and the role of AMPK/SIRT1-mediated autophagy during this process.

The protective effect of TSF on hepatic steatosis was verified in HFD-fed and MCDD-fed mice, two frequently used dietary models for NAFLD (Chung, 2017). HFD-fed mice form simple steatosis in the presence of lipid surplus but do not tend to progress in NASH spontaneously (Wang et al., 2016). During simple steatosis formation, HFD-fed mice exhibit a series of typical features of NALFD, such as obesity, hyperlipidemia, and insulin resistance (Mahana et al., 2016). TSF has been shown to directly improve hepatic steatosis, aberrant body weight gain, and serum lipid disorders and partially restore whole-body glucose metabolism by adjusting glucose tolerance, a well-known cause of NAFLD, thereby demonstrating the inhibitory effects of TSF on hepatic steatosis pathogenesis (Nagasawa et al., 2006; Zhou et al., 2015). MCDD-induced NASH involves the impairment of lipid efflux from hepatocytes through very low density lipoproteins (VLDL), leading to severe hepatic steatosis, inflammation, and a significant increase in serum transaminase activity within a short period (4–6 weeks) (Qiang et al., 2016; Tosello-Trampont et al., 2016). The MCDD model is one of the most well-established models for studying therapeutic strategies against hepatic inflammation progression (Machado et al., 2015; Mridha et al., 2017). Methionine and choline are precursors of phosphatidylcholine, the major constituent phospholipid of VLDL particles. VLDL is not secreted in the absence of methionine and choline, whereas TG and cholesterol are accumulated in the hepatocytes resulting in decreased serum TG and TC (Marcolin et al., 2011). Moreover, methionine is essential for adiposity, and methionine deficiency can lead to a reduction in white adipose tissue and to weight loss by accelerating lipolysis and enhancing metabolism (Rizki et al., 2006; Tanaka et al., 2014). Consistent with these reports, the MCDD-fed mice in the current study presented with decreased body weight and serum TG and TC levels and increased lipid accumulation in the liver. Moreover, TSF efficiently inhibited the increase in the levels of serum ALT and AST, hepatic steatosis, inflammatory cell infiltration, and necrosis *in situ* in MCDD-fed mice, exhibiting the strong hepatoprotective effect of TSF against oxidative hepatic injury resulting from lipid accumulation. Consistent with these results, in the current study, TSF exerted lipid-lowering effects and attenuated lipid accumulation in PA-stimulated HepG2 cells and primary mouse hepatocytes in a dose-dependent manner. In short, both *in vivo* and *in vitro* studies demonstrate the powerful effect of TSF on the improvement of hepatic steatosis.

Autophagy is critical for the maintenance of lipid homeostasis by hydrolyzing intracellular lipid droplets (Varshney et al., 2018). Portions of entire lipid droplets are wrapped in the autophagosome and decomposed into free fatty acid in the autolysosome, which is then followed by mitochondrial oxidation. Increasing evidence indicates that impaired autophagy is tightly associated with NAFLD development. Suppressing autophagy with 3-methyladenine, an inhibitor of autophagy initiation, or knocking out ATG5, a participant in the elongation process of autophagosome formation, leads to decreased lipid decomposition, free fatty acid β-oxidation, and subsequent intracellular lipid accumulation in free fatty acid-stimulated hepatocytes (Sun et al., 2018). Several *in vivo* studies also demonstrate that nutrient depletion activates autophagy and stimulates the binding of LC3B-II with lipid droplets while increasing the number of autophagosomes containing lipid droplets (Hur et al., 2015; Zhang et al., 2016). These observations suggest that autophagy mediates the decomposition of lipid droplets containing TG in mice liver, and pharmacological activation of hepatic autophagy may be a target for the treatment of NAFLD. Given that our previous results demonstrated the upregulation of autophagy by TSF in the kidney, we hypothesized that it has the potential to treat NAFLD by inducing hepatic autophagy (Zhao et al., 2017).

In the present study, we found that excessive lipid accumulation impaired hepatic autophagy in mice with NAFLD. In the liver of the HFD-fed or MCDD-fed mice, the levels of LC3B-II, a marker for autophagosome formation, were considerably reduced, whereas the levels of p62, a specific autophagy substrate that negatively correlates with autophagy activity, notably increased, thus indicating the suppression of autophagy. After TSF treatment, accumulation of hepatic LC3B-II and decrease in p62 were noted, demonstrating that TSF restored impaired autophagy in fatty livers. In addition, several important lines of evidence suggested that TSF-induced autophagy activation directly participated in the reduction of TG levels in the PA-stimulated HepG2 cells and primary mouse hepatocytes. First, TSF facilitated autophagy activation in steatotic hepatic cells in a dose-dependent manner. Second, TSF significantly reduced lipid accumulation and restored the impaired autophagy simultaneously in these cells. Third, the lysosomal functional inhibitor eliminated the effects of TSF on autophagy and lipid accumulation, indicating that TSF excited upstream regulators prior to the autolysosome pathway. Therefore, we concluded that TSF improved hepatic steatosis by autophagy activation and its related lipid decomposition. This finding provides a new mechanism for the lipid-lowering effect of TSF, in which autophagy plays an important role.

AMPK is the primary sensor of energy levels and could regulate energy homeostasis through autophagy (Song et al., 2015). AMPK is phosphorylated by the increase in the AMP/ATP ratio or other metabolic stressors and upregulates SIRT1 expression, thereby triggering autophagy (Sun et al., 2018). In the present study, TSF recovered phosphorylated AMPK in NAFLD mice and increased the ratio of p-AMPK/AMPK in steatotic hepatic cells in a dose-dependent manner, indicating that the AMPK-dependent pathway might participate in the TSF-induced activation of autophagy. Subsequently, CC blocked AMPK phosphorylation and the upregulation of SIRT1 in steatotic HepG2 cells treated with TSF, indicating that AMPK was involved in the upregulation of SIRT1 by TSF. SIRT1, a nicotinamide adenine dinucleotide (NAD)^+^-dependent deacetylase, modulates cellular metabolism and has emerged as a critical regulator of hepatic lipid homeostasis through autophagy (Yao et al., 2018). In the current study, we demonstrated that TSF restored the downregulated expression of SIRT1 due to NAFLD in mice. In addition, TSF-induced autophagy in a dose-dependent manner via SIRT1 expression in steatotic cells, as demonstrated by the abolishment of TSF-induced autophagy activation following siRNA-mediated SIRT1 knockdown. Interestingly, after SIRT1 expression and its mediated autophagy activation were abolished by gene regulation, the number of intracellular lipid droplets was not reduced by TSF, further demonstrating that SIRT1-mediated autophagy activation was responsible for the hepatic lipid clearance. For more convincing evidence of the beneficial effect of TSF against hepatic steatosis via SIRT1 induction, further studies are needed to investigate the metabolic changes in liver-specific SIRT1 knockout mice treated with vehicle or TSF. At present, this study provides ample evidence that TSF-induced hepatic steatosis remission is dependent on AMPK/SIRT1-mediated autophagy.

Regarding components in TSF, neohesperidin and calycosin-7-O-β-D-glucoside have been widely used to attenuate hepatic lipid accumulation in various rodent models of NAFLD (Jia et al., 2015; Duan et al., 2017; Wu, 2017). In addition, naringenin has been reported to reverse steatosis, expression of inflammatory factors, and infiltration of macrophages in the livers of rats fed with high-cholesterol diet over a long period of time (Chtourou et al., 2015). However, the underlying mechanisms of the hepatic lipid-lowering effects of these compounds remain unclear. According to the findings of the present study, we hypothesized that the activation of autophagy may be involved in their beneficial effects in NAFLD; however, this hypothesis needs to be explored in a future study.

## Conclusion

In conclusion, the present study demonstrated that autophagy was involved in relieving the effects of TSF against NAFLD, which were mediated by the AMPK/SIRT1 pathway (Figure 7D). These findings may improve our current understanding of the role of TSF in treating hepatic steatosis and provide an experimental basis for the clinical application of TSF in NAFLD and its related metabolic syndrome.

## Ethics Statement

This study was carried out in accordance with the recommendations in the Guide for the Care and Use of Laboratory Animals of the National Institutes of Health. The protocol was approved by the Ethics Committee of the China–Japan Friendship Hospital.

## Author Contributions

LP and PL designed the experiments. YW, ZS, QW, and YL performed the experiments. XL, TZ, NL, and NZ analyzed and interpreted the data. HZ and QL drafted the manuscript.

## Conflict of Interest Statement

The authors declare that the research was conducted in the absence of any commercial or financial relationships that could be construed as a potential conflict of interest.
