# Supplementary material for: Tangshen Formula Alleviates Hepatic Steatosis by Inducing Autophagy Through the AMPK/SIRT1 Pathway
Source: Front Physiol. 2019 Apr 26;10:494. doi: 10.3389/fphys.2019.00494 (PMC6498888; doi:10.3389/fphys.2019.00494)
Supplement: Supplementary file 1 [file Table_1.DOCX]

FIGURE S1


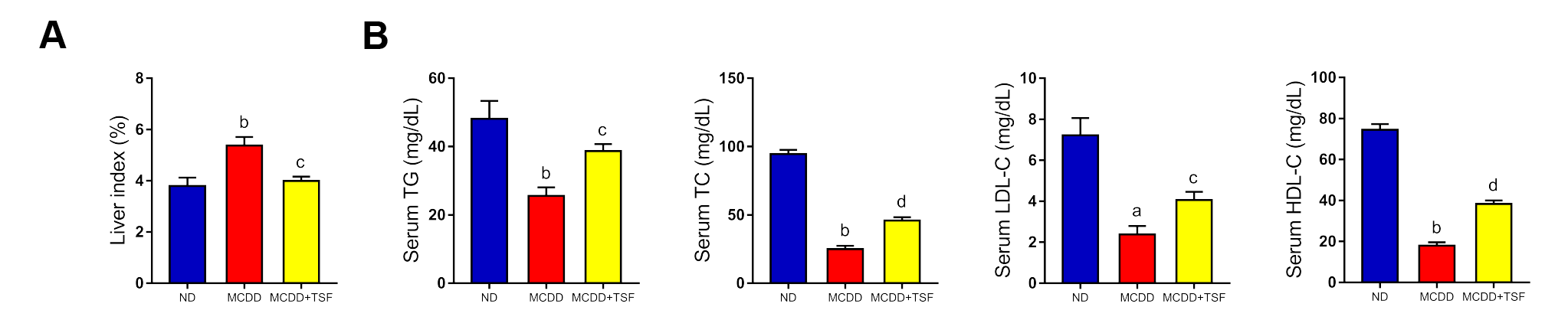


TSF improved liver index and serum disorders in mice fed a MCDD. (A) Liver index was calculated as the ratio of liver weight to body weight (%). (B) Serum TG, TC, LDL-C, and HDL-C levels in mice fed a MCDD were assayed with an automatic analyzer. Data are expressed as the mean ± SEM (n=6). ^a^ *P*< 0.05, ^b^ *P*< 0.01 vs. ND group; ^c^ *P* < 0.05, ^d^ *P*< 0.01 vs. MCDD group.

FIGURE S2


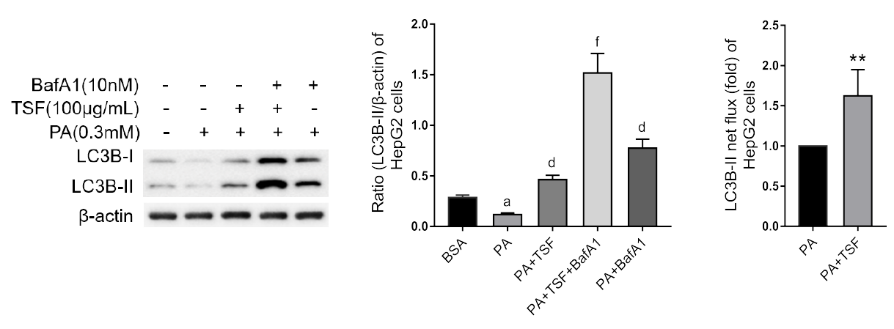


TSF alleviated steatosis through reinforcing autophagy in PA-stimulated HepG2 cells. Western blot assay and semi-quantitative analysis of LC3B-II of PA-stimulated HepG2 cells in response to TSF and BafA1 treatment for 24 h; LC3B-II net flux was assessed by subtracting the amount of LC3B-II in the absence of BafA1 from the amount of LC3B-II in the presence of BafA1 for each of the conditions; each LC3-II expression level was normalized by its β-actin expression level and the LC3B-II net flux of PA-stimulated cells was normalized to 1; data are expressed as the mean ± SEM of three independent experiments performed in triplicate. ^a^ *P*< 0.05 vs. BSA group; ^d^ *P*< 0.01 vs. PA group; ^f^ *P* < 0.01 vs. PA+TSF group; ^**^ *P* < 0.01 vs. PA+ BafA1 group.
